# Supplementary material for: The Effect of Alginate/Hyaluronic Acid Proportion on Semi-Interpenetrating Hydrogel Properties for Articular Cartilage Tissue Engineering
Source: Polymers (Basel). 2025 Feb 18;17(4):528. doi: 10.3390/polym17040528 (PMC11859035; doi:10.3390/polym17040528)
Supplement: Supplementary file 1 [file polymers-17-00528-s001.zip › polymers-3454498-supplementary.pdf]

The Effect of Alginate/Hyaluronic Acid Proportion on Semi-Interpenetrating Hydrogel Properties for Articular Cartilage Tissue Engineering †

Izar Gorroñogoitia [1,2], Sheila Olza [2,3,4], Ana Alonso-Varona [2] and Ane Miren Zaldua [1,*]

1 Leartiker S. Coop., 48270 Makina-Xemein, Spain; igorronogoitia@leartiker.com (I.G.)
2 Faculty of Medicine and Nursing, University of the Basque Country (UPV/EHU), 48940 Leioa, Spain; sheila.olza@ehu.eus (S.O.) and ana.alonsovarona@ehu.eus (A.A.-V.)
3 E2S UPPA, CNRS, IPREM, Universite de Pau et des Pays de l'Adour, 64600 Anglet, France
4 MANTA-Marine Materials Research Group, E2S UPPA, Universit'e de Pau et des Pays de l'Adour, 64600 Anglet, France
* Correspondence: amzaldua@leartiker.com; Tel.: +34-946-169-089
† This Article is a Revised and Expanded Version of a Conference Poster Entitled "The Effect of Alginate/Hyaluronic Acid Proportion on Semi-Ipn Hydrogel Properties for Articular Cartilage Tissue Engineering", which was presented at European Society for Biomaterials 2023 (ESB) at Davos (Switzerland) in September 4–8, 2023.

SUPLEMMENTARY INFORMATION:

Characterization of sodium alginate

Molecular weight was determined by gel permeation chromatography (GPC) with a pump, automatic injector, a precolumn (Ultrahydrogel guard column, Waters) together with 3 columns in series (Ultrahydrogel 2000, Ultrahydrogel 250, Ultrahydrogel 120, Waters), and a refractive index detector (Optilab T-Rex, Wyatt Technology). The mobile phase for the measurements was 0.1 M $NaNO_3$ at a flow rate of 0.6 mL/min, and tests were performed at 35 °C. The alginate sample was dissolved in the mobile phase at a concentration of 2 mg/mL and was injected in the columns. Data were analysed using ASTRA 6 software (Wyatt Technology). The distribution of molecular weight (MWD) was obtained from a calibration curve that was performed by injecting various polyethylene glycol (PEO) standards in the range of 410–1039000 Da.

Characterization of hyaluronic acid batches

When working with polymers from natural origin, the properties vary from batch to batch. This is crucial for the final properties of the hydrogel; therefore, it is very important to have an exhaustive control of the intrinsic properties of every batch. The molecular weight (M_w) of the hyaluronic acid (batches 230002+230005 and 230029+230056) was adjusted by combining two different extracts until obtaining very similar M_w to the first batch (210041) and measuring the viscosity with a rheometer, as shown in Figure S1. This allowed us to minimize the difference in properties as much as possible.

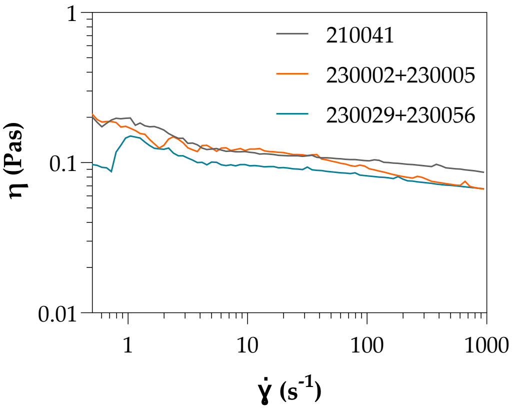

Figure S1. Viscosity curves for the hyaluronic acid batches at a concentration of % 2 (w/w) in PBS at 23 °C.

Rheological characterization

The flow behaviour index (n) was calculated using the Ostwald–de Waele model [1] shown in Equation S1, where τ is the shear stress (Pa), $\dot{\gamma}$ is the shear rate (s^{-1}), and parameters K and n are the flow consistency index and flow behaviour index.

$$\tau=K\dot{\gamma}^{n} \tag{S1}$$

Zero-shear viscosity (η_0) data were determined using the Carreau–Yasuda model [2] (Equation S2), where η is the shear viscosity, η_∞ is the infinite-shear viscosity, τ is the relaxation time of the fluid, $\dot{\gamma}$ the shear rate, n the flow index, and a is a constitutive parameter.

$$\eta=\eta_\infty+\frac{(\eta_0 - \eta_\infty)}{[1+(\tau\dot{\gamma})^a]^{\frac{1-n}{a}}} \tag{S2}$$

Table S1. Ostwald–de Waele parameters and zero-shear viscosity of alginate and hyaluronic acid single-component solutions at different concentrations and two different temperatures.

Formulation	T °C	*K* (Pas)	*n*	η_0 (Pas)
2Alg	23	3.86 ± 0.09	0.644 ± 0.005	1.18 ± 0.03
	37	2.65 ± 0.24	0.674 ± 0.008	0.80 ± 0.02
2HA	23	0.144 ±0.009	0.927 ± 0.010	0.161 ± 0.006
	37	0.101 ± 0.017	0.950 ± 0.024	0.115 ± 0.021
3Alg	23	18.29 ± 1.16	0.517 ± 0.001	5.08 ± 0.33
	37	12.99 ± 1.23	0.548 ± 0.008	3.24 ± 0.20
3GHA<br>3HA	23	0.69 ± 0.01	0.716 ± 0.001	0.42 ± 0.10
	37	0.50 ± 0.08	0.740 ± 0.019	0.32 ±0.06
4Alg	23	52.28 ± 1.02	0.438 ± 0.001	12.7 ± 0.20
	37	41.36 ± 0.40	0.440 ± 0.002	11.03 ± 0.66
4HA	23	1.216 ± 0.007	0.740 ± 0.005	0.658 ± 0.136
	37	0.933 ± 0.129	0.737 ± 0.007	0.601 ± 0.159

Table S2. Ostwald–de Waele parameters and zero-shear viscosity of alginate/hyaluronic acid hybrid solutions at different concentrations and two different temperatures.

Formulation	T °C	*K* (Pas)	*n*	η_0 (Pas)
2Alg1HA	23	6.69 ± 0.12	0.600 ± 0.002	2.04 ± 0.04
	37	5.00 ± 0.10	0.620 ± 0.002	1.43 ± 0.01
2Alg2HA	23	14.21 ± 0.73	0.537 ± 0.005	4.66 ± 0.32
	37	9.89 ± 0.42	0.564 ± 0.004	2.98 ± 0.08
3Alg1HA	23	30.45 ± 1.32	0.475 ± 0.008	8.58 ± 0.48
	37	21.59 ± 0.57	0.506 ± 0.006	5.15 ± 0.21
3Alg2HA	23	62.54 ± 4.50	0.418 ± 0.026	22.34 ± 0.89
	37	67.82 ± 4.01	0.443 ± 0.010	16.39 ± 3.54
4Alg1HA	23	66.97 ± 18.81	0.473 ± 0.067	23.10 ± 1.59
	37	57.80 ± 17.21	0.477 ± 0.038	16.31 ± 4.60
4Alg2HA	23	113.40 ± 9.24	0.355 ± 0.010	-
	37	110.32± 14.32	0.375 ± 0.003	-

Viscoelastic and mechanical characterization

Regarding the viscoelastic properties, as an example, the time-sweep experiment of alginate and its homologues at 2 % (w/w) after crosslinking with 100 mM of $CaCl_2$ is depicted in Figure S2, from which the storage modulus *G′* of all hydrogels was determined. It can be observed that the fully elastic behaviour of all hydrogels with storage moduli G′ (squares) is higher than the loss moduli *G′′* (triangles) across the whole time interval.

Concerning the mechanical properties, Figure S3 shows the stress–strain curve of alginate and its homologues at 2 % (w/w) obtained from unconfined compression experiments. Hydrogels' compression modulus was determined from these curves at a target strain of 10 % since all curves presented a non-linear behaviour.

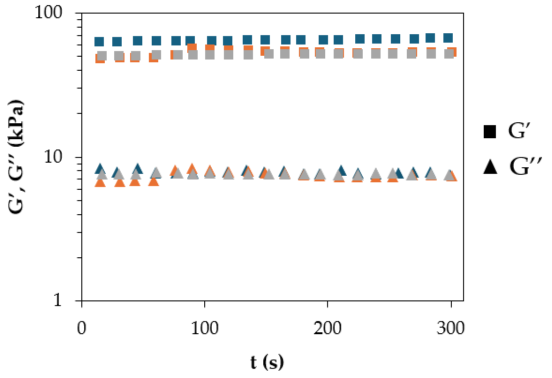

Figure S2. Storage modulus *G′* (squares) and loss modulus *G′′* (triangles) as a function of time for hydrogels: 2Alg (blue), 2Alg1HA (orange), and 2Alg2HA (grey) crosslinked with 100 mM of $CaCl_2$ at 37 °C (*n* = 3 per group).

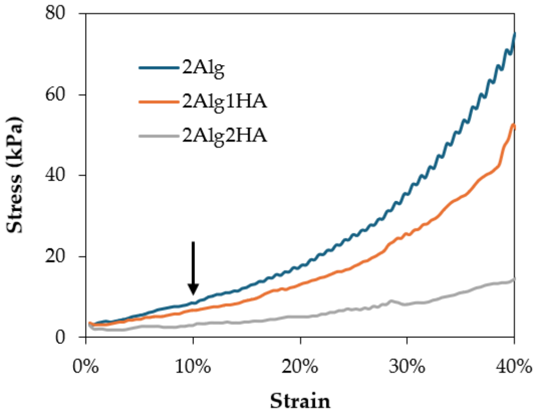

Figure S3. Stress–strain curves for alginate hydrogel at 2 % (w/w) and its homologues crosslinked with 100 mM of $CaCl_2$ at 37 °C (*n* = 5 per group).

Table S3. Values of the storage modulus *G′* and tangent modulus at 10 % strain for each alginate and its homologues crosslinked with 100 mM of $CaCl_2$.

Formulation	T °C	*G′* (kPa)	Tangent modulus %10 (kPa)
2Alg	23	74.94 ± 3.10	10.99 ± 1.46
	37	61.44 ± 2.34	8.80 ± 1.27
2Alg1HA	23	75.72 ± 3.00	8.31 ± 1.46
	37	56.23 ± 5.95	6.65 ± 0.30
2Alg2HA	23	60.26 ± 2.17	4.69 ± 0.58
			.69
	37	51.23 ± 0.57	3.05 ± 0.43

3Alg	23	147.10 ± 4.29	18.78 ± 0.83
	37	132.17 ± 6.69	16.07 ± 1.66
3Alg1HA	23	153.43 ± 9.14	17.93 ± 2.51
	37	137.39 ± 6.41	12.61 ± 1.61
3Alg2HA	23	132.71 ± 3.76	19.75 ± 2.49
	37	117.18 ± 3.25	14.01 ± 1.21
4Alg	23	194.70 ± 8.03	29.95 ± 2.81
	37	189.79 ± 6.24	23.13 ± 4.34
4Alg1HA	23	179.34 ± 5.29	26.46 ± 5.79
	37	165.27 ± 3.61	15.66 ± 1.94
4Alg2HA	23	175.78 ± 9.37	26.31 ± 4.28
	37	152.59 ± 9.55	13.03 ± 2.43

Printability evaluation

The quantitative evaluation of the printed structures was performed by measuring the filament diameter (D_{exp}), the area of pores and the perimeter of pores. Theoretical target dimensions are expressed below the table.

Table S4. Printability parameters for all printed hydrogels.

Sample	$CaCl_2$ (mM)	D_{exp} (mm)	Perimeter (mm)	Area (mm^2)
2Alg	Uncrosslinked	1.47 ± 0.07	12.17 ± 0.60	10.69 ± 0.83
	100	1.09 ± 0.27	13.27 ± 0.36	12.75 ± 0.85
2Alg1HA	Uncrosslinked	1.25 ± 0.02	13.60 ± 0.24	13.23 ± 0.45
	100	1.09 ± 0.12	13.17 ± 0.57	12.31 ± 1.32
2Alg2HA	Uncrosslinked	1.38 ± 0.02	12.94 ± 0.26	12.12 ± 0.32
	100	1.15 ± 0.03	12.56 ± 0.83	11.49 ± 1.63
3Alg	Uncrosslinked	1.63 ± 0.04	12.18 ± 0.23	10.89 ± 0.20
	100	1.00 ± 0.15	13.51 ± 0.25	13.27 ± 0.75
3Alg1HA	Uncrosslinked	1.27 ± 0.02	13.62 ± 0.27	13.20 ± 0.40
	100	1.31 ± 0.02	13.29 ± 0.21	12.92 ± 0.39
3Alg2HA	Uncrosslinked	1.19 ± 0.05	14.35 ± 0.17	14.39 ± 0.30
	100	0.95 ± 0.04	13.27 ± 0.03	12.43 ± 0.21
4Alg	Uncrosslinked	1.43 ± 0.09	13.26 ± 0.42	12.44 ± 0.70
	100	0.78 ± 0.05	13.83 ± 0.71	13.93 ± 1.41
4Alg1HA	Uncrosslinked	1.21 ± 0.04	14.28 ± 0.04	14.31 ± 0.32
	100	1.27 ± 0.03	13.89 ± 0.14	13.82 ± 0.27
**Intended dimension parameters: perimeter = 20 mm; area = 25 mm**				

References

1. Yeung, R.A.; Kennedy, R.A. A Comparison of Selected Physico-Chemical Properties of Calcium Alginate Fibers Produced Using Two Different Types of Sodium Alginate. *J. Mech. Behav. Biomed. Mater.* **2019**, *90,* 155–164. https://doi.org/10.1016/j.jmbbm.2018.10.011.
2. Dodero, A.; Vicini, S.; Alloisio, M.; Castellano, M. Sodium Alginate Solutions: Correlation between Rheological Properties and Spinnability. *J Mater Sci* **2019**, *54*, 8034–8046, doi:10.1007/s10853-019-03446-3.
